# Bilateral neurotrophic keratitis associated with Gilteritinib therapy in a patient with acute myeloid leukemia: a case report

**DOI:** 10.22336/rjo.2025.95

**Published:** 2025

**Authors:** Mihai-Luca Cioboată, Suher Abduraman, Bogdana Maliș, Radu Burcea, Miruna Cioboată

**Affiliations:** 1 “Prof. Dr. Mircea Olteanu” Clinical Institute of Ophthalmological Emergencies, Bucharest, Romania; 2 “Carol Davila” University of Medicine and Pharmacy, Bucharest, Romania

**Keywords:** neurotrophic keratitis, Gilteritinib, FLT3 inhibitor, acute myeloid leukemia, ocular toxicity, TKI = tyrosine kinase inhibitor, AML = acute myeloid leukemia, FLT3 = FMS-like tyrosine kinase 3, ITD = internal tandem duplication, TKD = tyrosine kinase domain, EGFR = epidermal growth factor receptor

## Abstract

**Introduction:**

We report the first documented case of bilateral neurotrophic keratitis potentially associated with Gilteritinib therapy.

**Case Presentation:**

A 52-year-old male patient presented with bilateral blurred vision and photophobia due to neurotrophic keratitis, which developed after two years of treatment with Gilteritinib - a second-generation tyrosine kinase inhibitor (TKI) used for relapsed or refractory acute myeloid leukemia (AML) with a confirmed FMS-like tyrosine kinase 3 (FLT3) mutation. His best-corrected visual acuity (BCVA) was 20/100 in the right eye and 20/40 in the left eye. Biomicroscopic examination revealed persistent epithelial defects, reduced corneal sensitivity, and no tear-film abnormalities or eyelid pathology. Gilteritinib was discontinued in consultation with the hematology team, and topical insulin with preservative-free lubricants was initiated. Within one week, the epithelial defects had healed, and at one month, visual acuity had improved to 20/20 in the left eye and 20/32 in the right eye, with residual central leukoma.

**Discussion:**

Neurotrophic keratitis is a rare corneal disorder caused by impaired innervation and defective epithelial healing. After exclusion of all known etiologies, prolonged Gilteritinib therapy was considered the most likely cause in our patient, possibly due to off-target effects on pathways involved in corneal nerve and epithelial homeostasis. Treatment with artificial tears and topical insulin led to favorable epithelial healing, underscoring the need for awareness of potential ocular surface toxicity with newer tyrosine kinase inhibitors.

**Conclusion:**

This case highlights potential ocular neurotoxicity associated with Gilteritinib, a targeted therapy not previously linked to corneal nerve dysfunction. Increased clinical awareness is recommended for ophthalmologists and hematologists managing patients with FLT3-mutated AML who are receiving targeted therapies.

## Introduction

Acute myeloid leukemia (AML) is an aggressive bone marrow disorder caused by genetic mutations in hematopoietic stem cells, leading to excessive production of abnormal clonal myeloid precursor cells [**1**]. The disease most commonly affects older adults and exhibits substantial genetic and molecular heterogeneity, which has important diagnostic, prognostic, and therapeutic implications. FMS-like tyrosine kinase 3 (FLT3) gene mutations are identified in about 30% of newly diagnosed AML cases. The presence of FLT3-internal tandem duplication (ITD) mutations is associated with an unfavorable prognosis, characterized by shorter overall survival and relapse-free survival [**2-4**].

Gilteritinib is a highly selective inhibitor that targets both the ITD and tyrosine kinase domain (TKD) mutations of the FLT3 receptor [**5**]. It is the first second-generation tyrosine kinase inhibitor (TKI) approved for the treatment of patients with relapsed or refractory AML harboring an FLT3 mutation. Based on the results of the Phase III ADMIRAL trial, Gilteritinib received regulatory approval for use in patients with relapsed or refractory FLT3-mutated AML, demonstrating significantly improved overall survival compared with standard chemotherapy [**5,6**]. While Gilteritinib is generally well tolerated, its adverse effect profile includes fatigue, elevated liver transaminases, noninfectious diarrhea, nausea, constipation, musculoskeletal pain, dyspnea, edema, rash, pneumonia, stomatitis, cough, headache, dizziness, hypotension, and fever [**7**]. Ocular toxicities are rarely described in association with Gilteritinib or other FLT3 inhibitors. Reported ocular events include dry eye in approximately 6.3% of patients [**8**], retinal hemorrhages in 7.7% [**9**], and only a single case of acute macular neuroretinopathy documented to date [**10**].

To the best of our knowledge, bilateral neurotrophic keratitis associated with Gilteritinib therapy has not been previously documented in the literature. We herein report a case of bilateral neurotrophic keratitis and describe its management in a patient with relapsed AML undergoing treatment with Gilteritinib.

## Case presentation

**Fig. 1:**
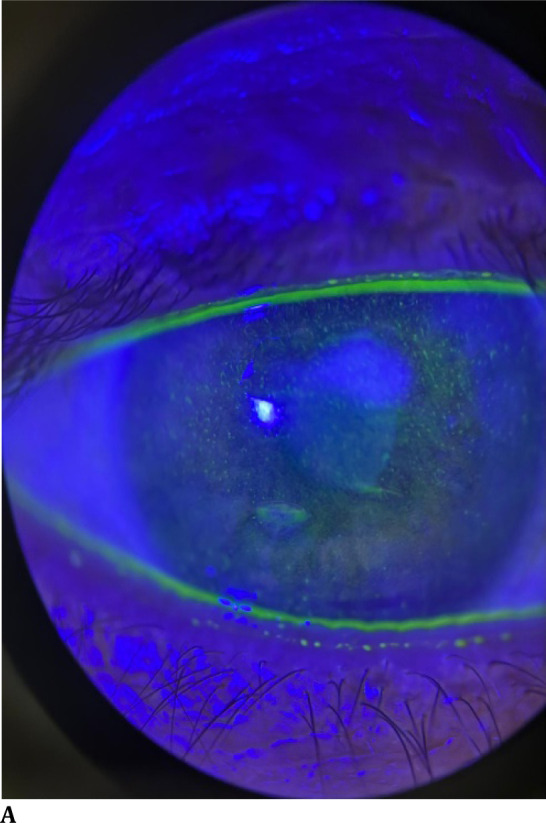

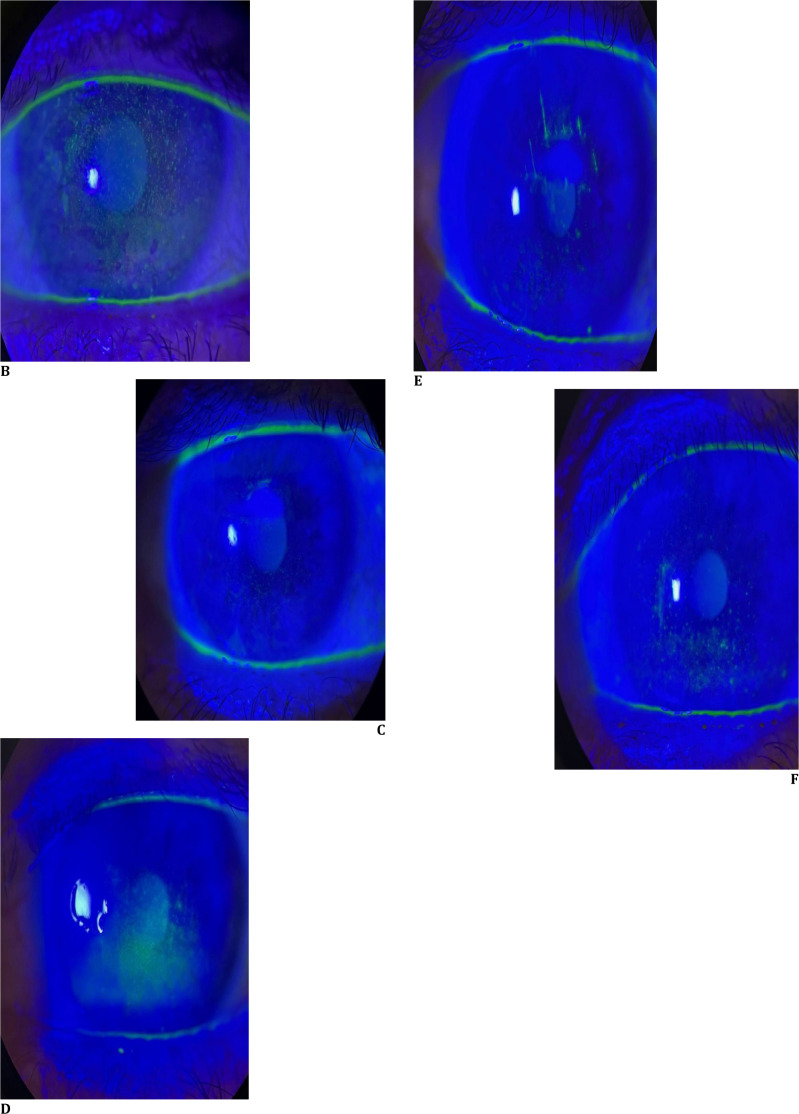
Representative images of both eyes treated with topical insulin at baseline (**A, B**), at 1 week (**C, D**), and at 1 month (**E, F**), respectively

A 52-year-old male patient presented to our department with bilateral blurred vision and photophobia, which had progressively worsened over the past few weeks. His medical history was significant for AML with an FLT3-ITD mutation, diagnosed four years prior and initially treated with cytarabine and allogeneic hematopoietic stem cell transplantation. The patient experienced a relapse eighteen months post-transplant and subsequently received Gilteritinib (XOSPATA®, Astellas Pharma US, Inc., Northbrook, Illinois) at a dose of 120 mg/day for two years. On examination, his best-corrected visual acuity (BCVA) was 20/100 in the right eye and 20/40 in the left eye. Slit-lamp biomicroscopy revealed punctate epithelial fluorescein staining and persistent epithelial defects (PED) with smooth, rolled edges in both eyes, and stromal opacity in the right eye (**Fig. 1A, B**). Corneal sensitivity was assessed using a Cochet-Bonnet esthesiometer (Luneau Ophthalmics, Pont-de-l’Arche, France), averaging values across five corneal sectors. Both eyes demonstrated reduced corneal sensation, with mean measurements of 10 mm in the right eye and 15 mm in the left eye. No abnormalities were noted in the eyelids, with no signs of malposition, lagophthalmos, or blepharitis that could potentially compromise the ocular surface. Tear film assessment demonstrated normal tear breakup time, effectively ruling out evaporative dry eye disease, and Schirmer test results were within normal limits, indicating adequate tear production. There were no signs of infection, and there was no anterior chamber inflammation. The patient was diagnosed with bilateral neurotrophic keratitis, stage 2, according to Mackie’s classification. Treatment was initiated with topical insulin and preservative-free artificial tears, and Gilteritinib therapy was promptly discontinued in consultation with the treating hematologists. At the one-week follow-up, the clinical course was favorable, with resolution of the punctate epitheliopathy and healing of the PED (**Fig. 1C, D**). The patient was continued on topical insulin in combination with lubricating agents. At the one-month assessment, BCVA measured 20/32 in the right eye and 20/20 in the left eye. Slit-lamp biomicroscopy revealed a central corneal leukoma extending over approximately one-half of the pupillary area (**Fig. 1E, F**).

## Discussion

Neurotrophic keratitis is a rare, degenerative corneal disease caused by impaired corneal innervation, resulting in reduced corneal sensitivity and defective epithelial healing. The most common etiologies include herpetic keratitis, ocular surgical procedures, diabetes mellitus, chemical burns, chronic use of topical ocular medications, and intracranial tumors or trauma involving the trigeminal nerve [**11**]. In our patient, all known potential etiologies were systematically excluded, and we believe that the development of neurotrophic keratitis was most likely related to prolonged Gilteritinib therapy. Although neurotrophic keratitis has not been previously reported with FLT3 inhibitors, off-target effects on tyrosine kinases involved in neuronal or epithelial homeostasis could theoretically impair corneal nerve function and epithelial maintenance.

Reviewing the literature, we identified only one previously reported case of neurotrophic keratitis linked to Gefitinib, a selective epidermal growth factor receptor (EGFR) TKI. The patient developed bilateral corneal ulcers that progressed to perforation despite treatment and was managed with bilateral penetrating keratoplasty [**12**]. Experimental studies in rats have shown that treatment with EGFR inhibitors can lead to thinning of the corneal epithelium, a dose-dependent delay in wound healing, and reduced epithelial cell proliferation, which may explain the ocular surface complications observed in clinical practice [**13**]. In our case, Gilteritinib represents a relatively new therapeutic option, and its full spectrum of potential effects- particularly on the ocular surface- remains to be determined, highlighting the need for ongoing clinical vigilance and further experimental research.

Management options for neurotrophic keratitis range from supportive therapies such as artificial tears, therapeutic contact lenses, and autologous serum tears to surgical interventions, including temporary or permanent tarsorrhaphy, botulinum toxin injection into the levator muscle, amniotic membrane transplantation, conjunctival flap procedures, and corneal transplantation. Novel therapies include cenegermin, topical insulin, matrix regeneration therapy, plasma rich in growth factors, thymosin β4, nicergoline, and corneal neurotization [**11,14,15**]. In our patient, we recommended artificial tears and topical insulin, resulting in a favorable outcome.

Topical insulin has been shown to facilitate epithelial proliferation and wound healing by activating insulin and IGF-1 receptors, thereby promoting epithelial migration and mitotic activity. Current evidence indicates that topical insulin is safe and well-tolerated, though long-term studies are needed to evaluate its safety and potential delayed side effects [**16**]. To date, no studies have directly compared topical insulin with cenegermin, a recombinant human nerve growth factor, although cenegermin has consistently shown sustained efficacy with longer follow-up [**17,18**]. Topical insulin demonstrated superior epithelialization outcomes compared with autologous serum and may therefore be considered a potential first-line therapy [**19**]. In our case, cenegermin was not commercially available, and autologous serum was contraindicated due to the patient’s hematologic condition.

## Conclusion

This case highlights potential ocular neurotoxicity associated with Gilteritinib, a targeted therapy not previously associated with corneal nerve dysfunction. Heightened clinical vigilance is warranted among ophthalmologists and hematologists treating patients with targeted therapies for FLT3-mutated AML.

## References

[1] Pelcovits A, Niroula R (2020). Acute Myeloid Leukemia: A Review. R I Med J (2013).

[2] Ley TJ, Miller C, Ding L, Raphael BJ, Mungall AJ, Robertson A, Hoadley K, Triche TJ, Laird PW, Baty JD, Fulton LL, Fulton R, Heath SE, Kalicki-Veizer J, Kandoth C, Klco JM, Koboldt DC, Kanchi KL, Kulkarni S, Lamprecht TL, Larson DE, Lin L, Lu C, McLellan MD, McMichael JF, Payton J, Schmidt H, Spencer DH, Tomasson MH, Wallis JW, Wartman LD, Watson MA, Welch J, Wendl MC, Ally A, Balasundaram M, Birol I, Butterfield Y, Chiu R, Chu A, Chuah E, Chun HJ, Corbett R, Dhalla N, Guin R, He A, Hirst C, Hirst M, Holt RA, Jones S, Karsan A, Lee D, Li HI, Marra MA, Mayo M, Moore RA, Mungall K, Parker J, Pleasance E, Plettner P, Schein J, Stoll D, Swanson L, Tam A, Thiessen N, Varhol R, Wye N, Zhao Y, Gabriel S, Getz G, Sougnez C, Zou L, Leiserson MD, Vandin F, Wu HT, Applebaum F, Baylin SB, Akbani R, Broom BM, Chen K, Motter TC, Nguyen K, Weinstein JN, Zhang N, Ferguson ML, Adams C, Black A, Bowen J, Gastier-Foster J, Grossman T, Lichtenberg T, Wise L, Davidsen T, Demchok JA, Shaw KR, Sheth M, Sofia HJ, Yang L, Downing JR, Eley G, Cancer Genome Atlas Research Network (2013). Genomic and epigenomic landscapes of adult de novo acute myeloid leukemia. N Engl J Med.

[3] Welch JS, Ley TJ, Link DC, Miller CA, Larson DE, Koboldt DC, Wartman LD, Lamprecht TL, Liu F, Xia J, Kandoth C, Fulton RS, McLellan MD, Dooling DJ, Wallis JW, Chen K, Harris CC, Schmidt HK, Kalicki-Veizer JM, Lu C, Zhang Q, Lin L, O’Laughlin MD, McMichael JF, Delehaunty KD, Fulton LA, Magrini VJ, McGrath SD, Demeter RT, Vickery TL, Hundal J, Cook LL, Swift GW, Reed JP, Alldredge PA, Wylie TN, Walker JR, Watson MA, Heath SE, Shannon WD, Varghese N, Nagarajan R, Payton JE, Baty JD, Kulkarni S, Klco JM, Tomasson MH, Westervelt P, Walter MJ, Graubert TA, DiPersio JF, Ding L, Mardis ER, Wilson RK (2012). The origin and evolution of mutations in acute myeloid leukemia. Cell.

[4] Daver N, Venugopal S, Ravandi F (2021). FLT3 mutated acute myeloid leukemia: 2021 treatment algorithm. Blood Cancer J.

[5] Loschi M, Sammut R, Chiche E, Cluzeau T (2021). FLT3 Tyrosine Kinase Inhibitors for the Treatment of Fit and Unfit Patients with FLT3-Mutated AML: A Systematic Review. Int J Mol Sci.

[6] Gupta SV, Jose N, Tafuto B (2024). The Impact of Gilteritinib on Overall Survival of Adult Patients with FLT3 Positive Acute Myeloid Leukemia: A Systematic Review. Princ Pract Clin Res.

[7] (2019). Xospata Prescribing Information.

[8] Vitiello L, Lixi F, Coco G, Giannaccare G (2024). Ocular Surface Side Effects of Novel Anticancer Drugs. Cancers (Basel).

[9] Lixi F, Giannaccare G, Salerno G, Gagliardi V, Pellegrino A, Vitiello L (2024). Side Effects of Novel Anticancer Drugs on the Posterior Segment of the Eye: A Review of the Literature. J Pers Med.

[10] Liu Y, Haq Z, Pasricha ND, Bever GJ (2020). Acute Macular Neuroretinopathy Associated With an Oral FLT3 Inhibitor. JAMA Ophthalmol.

[11] NaPier E, Camacho M, McDevitt TF, Sweeney AR (2022). Neurotrophic keratopathy: current challenges and future prospects. Ann Med.

[12] Gozzi F, Tiseo M, Facchinetti F, Gandolfi S, Rubino P (2021). Bilateral Severe Corneal Ulcer in a Patient with Lung Adenocarcinoma Treated with Gefitinib. Case Rep Ophthalmol.

[13] Nakamura Y, Sotozono C, Kinoshita S (2001). The epidermal growth factor receptor (EGFR): role in corneal wound healing and homeostasis. Exp Eye Res.

[14] Di Zazzo A, Coassin M, Varacalli G, Galvagno E, De Vincentis A, Bonini S (2019). Neurotrophic keratopathy: Pros and cons of current treatments. Ocul Surf.

[15] Ong ES, Jeng BH (2021). Current and future therapies for persistent corneal epithelial defects and neurotrophic keratopathy. Curr Opin Ophthalmol.

[16] Scripcă R, Istrate S, Ungureanu E, Oprea Ș, Anton N, Boruga M, Moga MA, Onofrei AG (2025). The Therapeutic Potential of Insulin Eye Drops in Neurotrophic Keratopathy: A Comprehensive Review. Biomedicines.

[17] Pflugfelder SC, Massaro-Giordano M, Perez VL, Hamrah P, Deng SX, Espandar L, Foster CS, Affeldt J, Seedor JA, Afshari NA, Chao W, Allegretti M, Mantelli F, Dana R (2020). Topical Recombinant Human Nerve Growth Factor (Cenegermin) for Neurotrophic Keratopathy: A Multicenter Randomized Vehicle-Controlled Pivotal Trial. Ophthalmology.

[18] Bonini S, Lambiase A, Rama P, Sinigaglia F, Allegretti M, Chao W, Mantelli F, REPARO Study Group (2018). Phase II Randomized, Double-Masked, Vehicle-Controlled Trial of Recombinant Human Nerve Growth Factor for Neurotrophic Keratitis. Ophthalmology.

[19] Diaz-Valle D, Burgos-Blasco B, Rego-Lorca D, Puebla-Garcia V, Perez-Garcia P, Benitez-Del-Castillo JM, Herrero-Vanrell R, Vicario-de-la-Torre M, Gegundez-Fernandez JA (2022). Comparison of the efficacy of topical insulin with autologous serum eye drops in persistent epithelial defects of the cornea. Acta Ophthalmol.

